# Selective Laser Sintering of Atomoxetine Tablets: An Innovative Approach for Small-Scale, Personalized Production

**DOI:** 10.3390/pharmaceutics17060794

**Published:** 2025-06-18

**Authors:** Gordana Stanojević, Ivana Adamov, Snežana Mugoša, Veselinka Vukićević, Svetlana Ibrić

**Affiliations:** 1 Institute for Medicines and Medical Devices of Montenegro, Ivana Crnojevića 64a, 81000 Podgorica, Montenegro; snezana.mugosa@cinmed.me (S.M.); veselinka.vukicevic@cinmed.me (V.V.); 2 Faculty of Medicine, University of Montenegro, Kruševac bb, 81000 Podgorica, Montenegro; 3 Department of Pharmaceutical Technology and Cosmetology, Faculty of Pharmacy, University of Belgrade, Vojvode Stepe 450, 11221 Belgrade, Serbia; ivana.adamov@pharmacy.bg.ac.rs (I.A.); svetlana.ibric@pharmacy.bg.ac.rs (S.I.)

**Keywords:** three-dimensional (3D) printing, additive manufacturing, selective laser sintering (SLS), personalized therapy, atomoxetine, tablet formulation

## Abstract

**Background/Objectives:** The growing interest in personalized medicine has accelerated the exploration of three-dimensional (3D) printing technologies in pharmaceutical applications. This study investigates the potential of selective laser sintering (SLS) as a flexible, small-scale manufacturing method for atomoxetine tablets tailored for individualized therapy, comparing it with conventional direct compression. **Methods:** Atomoxetine tablets were produced using SLS 3D printing with varying laser scanning speeds and compared to tablets made via a compaction simulator. Formulations were based on hydroxypropyl methylcellulose (HPMC) as the primary matrix former. The physical properties, drug content, disintegration time, and dissolution profiles were evaluated. The structural and chemical integrity were assessed using SEM, FTIR, DSC, and XRPD. **Results:** The SLS tablets exhibited comparable mechanical properties and drug content to those made by compaction. Lower laser speeds produced harder tablets with slower disintegration, while higher speeds yielded more porous tablets with ultra-rapid drug release (>85% in 15 min). All tablets met the European Pharmacopoeia dissolution criteria. No significant drug–excipient interactions or changes in crystallinity were detected. **Conclusions:** SLS printing is a viable alternative to traditional tablet manufacturing, offering control over drug release profiles through parameter adjustment. The technique supports the development of high-quality, patient-specific dosage forms and shows promise for broader implementation in personalized pharmaceutical therapy.

## 1. Introduction

The suboptimal therapeutic outcomes associated with traditional, one-size-fits-all medicines have driven the pharmaceutical industry to embrace personalized medicine, which aims to tailor therapies in dosage and composition to better address individual patient needs [1]. Personalized medicine refers to the customization of medical treatment based on individual characteristics such as genetics, age, weight and comorbidities. However, the production of personalized medicines using conventional manufacturing methods remains both costly and inefficient [2]. In response to these challenges, advanced manufacturing technologies such as three-dimensional (3D) printing, have emerged as promising solutions.

Three-dimensional printing, a form of additive manufacturing, is transforming the pharmaceutical industry by enabling the fabrication of patient-specific therapies rather than relying on mass production with limited dosing flexibility [3]. By employing a layer-by-layer fabrication approach based on predefined digital designs, this technology enables the rapid production of complex dosage forms that are difficult or impossible to achieve with traditional manufacturing techniques. Additionally, it allows for the efficient production of small batches customized for individual patients [4,5,6]. Since the introduction of Spritam^®^, the first FDA-approved 3D-printed medicine, the field has seen remarkable progress. Cutting-edge research has revealed a broad range of innovative applications, inspiring continued exploration into the unique advantages 3D printing offers for pharmaceutical development [3].

Despite its clear benefits, the adoption of 3D printing in healthcare remains limited, largely due to the absence of specific, dedicated regulatory frameworks. Namely, comprehensive regulatory guidelines for 3D-printed pharmaceuticals are needed. As the technology matures and research progresses, it is expected to pave the way for robust scientific standards, facilitating its practical and regulated application in the pharmaceutical industry [7]. Quality requirements for 3D-printed tablets have not yet been clearly defined. While the European Pharmacopoeia (EP) provides quality testing standards for conventional tablets, it remains uncertain whether these standards are fully applicable to 3D-printed tablets. To enable the clinical adoption and safe use of this emerging technology, it is essential to address and clarify these quality requirements [8].

Various 3D printing techniques, such as fused deposition modeling (FDM), binder and material jetting, semi-solid extrusion (SSE), stereolithography (SLA) and selective laser sintering (SLS), have been explored so far for pharmaceutical applications [9,10,11,12,13]. Among them, SLS has emerged as particularly promising for fabricating oral dosage forms. It constructs objects directly from powder by selectively fusing particles with localized laser heating. The process involves layer-by-layer construction, where a laser draws a pattern on a powder bed, and subsequent layers are added and fused. This method primarily uses carbon dioxide lasers to fuse powder particles into the desired structures. SLS technique has gained recognition in pharmaceutical applications due to its scalability and straightforward process [14,15].

Notably, SLS offers several advantages, including high resolution, powder recyclability, and a solvent-free process that eliminates the need for additional solvent removal steps [16,17]. This technique is highly versatile, capable of producing tablets with tailored drug release profiles, ranging from immediate to sustained release. These characteristics depend on the formulation composition and process parameters such as the laser scanning speed, power, and layer thickness [17,18]. Moreover, SLS is particularly effective in producing porous, fast-dissolving dosage forms, such as orodispersible tablets, which are difficult to achieve with other additive manufacturing methods. Importantly, SLS allows the use of commonly employed pharmaceutical excipients that are conventionally used in tablet manufacturing, facilitating easier formulation development and regulatory acceptability.

Due to its powder-based nature, SLS produces tablets that closely resemble the traditionally compressed ones in appearance and texture, which may enhance patient acceptance compared to other 3D printing methods [19]. However, currently available SLS printers are not specifically designed for pharmaceutical use. Despite this limitation, extensive research has focused on SLS for oral dosage forms, with its application in pharmaceuticals being proposed as early as the late 1990s. Since 2017, when SLS was first used to produce drug-loaded oral tablets [20], the number of publications on this topic has significantly increased. These studies cover diverse areas, including the development of complex porous structures for controlled drug release [21] as well as the influence of various printing parameters on the properties of the final dosage forms [17,22,23]. Because SLS was originally developed for industrial purposes, adapting it for pharmaceutical use is a complex and iterative process that heavily depends on user expertise for successful outcomes [24].

On the other hand, numerous studies have investigated the potential of SLS for the production of pharmaceuticals in small batch sizes and confirmed the important role of 3D printing in the concept of precision medicine and so-called on-demand therapy [3,7,16]. Atomoxetine, a selective norepinephrine reuptake inhibitor widely prescribed for the treatment of attention-deficit/hyperactivity disorder (ADHD), was considered a suitable model drug for the individualization of therapy [25]. Since atomoxetine is administered in a wide therapeutic dose range, usually from 10 mg to 100 mg per day, depending on the patient’s body weight, age and metabolic status, and due to the limited availability of dosage forms on the market (capsules only), atomoxetine exhibits all the characteristics of a suitable candidate to be investigated in the context of demand-led therapy.

Therefore, the aim of this study was to investigate the feasibility of using the SLS technique to produce atomoxetine tablets with optimized mechanical and pharmaceutical properties and at the same time to evaluate their suitability for the treatment of ADHD. The research focuses on identifying the optimal formulation composition and printing parameters to produce tablets with robust mechanical properties and desirable pharmaceutical-technological characteristics. For comparative purposes, tablets of identical composition were also manufactured using a compaction simulator to mimic traditional direct compression methods. This comparison aims to evaluate the potential of SLS as a viable alternative to direct compression, particularly in the context of small-scale, patient-specific manufacturing versus large-scale industrial production.

## 2. Materials and Methods

### 2.1. Materials

Atomoxetine hydrochloride (Ph. Eur. 11.0, Hemofarm AD, Vršac, Serbia) was selected as the model drug, while Candurin^®^ Gold Sheen (Merck, Darmstadt, Germany) was added to improve the laser energy absorption. The formulations for testing SLS printing, prepared with variations in process parameters, included the following excipients:

- Mannitol Parteck^®^ M 200 (Merck, Darmstadt, Germany);
- Kollidon^®^ VA 64 Fine (vinylpyrrolidone-vinyl acetate copolymer, BASF, Ludwigshafen, Germany);
- Hydroxypropyl methylcellulose (HPMC, Vivapharm^®^ E3, JRS Pharma, Rosenberg, Germany);
- Sodium stearyl fumarate (Merck, Darmstadt, Germany);
- Croscarmellose sodium (Sigma-Aldrich, Darmstadt, Germany);
  and directly compressible, co-processed excipients:
- Retalac^®^ (Meggle, Wasserburg am Inn, Germany), consisting of 50% lactose monohydrate and 50% HPMC;
- Starlac^®^ (Meggle, Wasserburg am Inn, Germany), consisting of 85% lactose monohydrate and 15% maize starch.

### 2.2. Methods

#### 2.2.1. Preparation of the Mixture for Tablet Production

The formulations were prepared with a fixed atomoxetine content of 10% and supplemented with 3% Candurin^®^ Gold Sheen to enhance laser energy absorption and improve printability. A number of polymers used as main components of pharmaceutical compositions for SLS 3D printing are not able to absorb the laser light sufficiently; therefore, the addition of a small amount of laser absorbers is required [16]. Candurin^®^ Gold Sheen is often used as a photoabsorber in pharmaceutical powder mixtures as an approved pharmaceutical excipient [10,16]. Fina et al. suggested that 3% Candurin^®^ Gold Sheen is optimal to improve the optical properties of the formulations consisting mainly of Kollicaot IR and Eudragit L100-55 due to its good absorption at 445 nm, which also corresponds to the printer’s blue diode laser. The powder particles in the presence of Candurin^®^ Gold Sheen efficiently absorb the energy of the laser light and thus also ensure a successful sintering process [20]. The specific compositions of the formulations, along with the applied process parameters are detailed in Table 1.

Each formulation was prepared by weighing 100 g of the mixture, consisting of the active pharmaceutical ingredient and excipients, followed by blending in a powder mixer (Farmalabor, Canosa di Puglia, Italy) for 10 min at a speed of 60 rpm.

#### 2.2.2. Tablet Production Using the SLS Technique

Three-dimensional tablet models with dimensions of 8 mm × 3 mm were designed using Autodesk Fusion 360 software, version 2.0.8809 (Autodesk Inc., San Francisco, CA, USA). The 3D tablet models were exported in .STL format and imported into Sintratec Central software version 1.2.0 (Sintratec, Brugg, Switzerland). Printing was performed on the Sintratec Kit printer (Sintratec, Brugg, Switzerland). Technical modifications were made to the printer’s reservoir and chamber to adapt the Sintratec Kit SLS printer for tablet production. To minimize the required quantity of the mixture, the chamber and reservoir were narrowed. For each printing cycle, 100 g of the prepared mixture was used. The printing parameters were controlled using the Sintratec Central software.

For the prepared formulations, initial printing tests were conducted by applying different process conditions, including varying the powder surface temperature, chamber temperature, and laser scanning speeds. Based on these tests, the optimal parameters and formulations for successful printing were identified. The applied printing conditions are summarized in Table 1. The hatch spacing value was set to 250 µm, while the layer thickness was kept constant at 100 µm. According to the data available in the literature, to ensure adequate transfer of the mixture from the reservoir to the printing chamber, the chamber temperature was set to be slightly below the powder surface temperature [26].

#### 2.2.3. Tablet Production Using a Compaction Simulator

Tablet production using a compaction simulator was performed with the formulation that demonstrated the most successful printing outcomes and desirable tablet characteristics during the initial SLS experiments involving various excipients and process conditions. The tablet mixture was compressed using the Gamlen compaction simulator, software version 3.26.0.0 (Gamlen Tabletting, London, UK) into round tablets with a diameter of 6 mm and a mass of 100 mg. The compaction speed was kept constant (60 mm/min). The tablet mixture was compressed using a compaction pressure of 300 MPa.

#### 2.2.4. Tablet Characterization Methods

Physical and Mechanical Properties

Ten tablets from each formulation of SLS printed tablets were weighed using an analytical balance (Kern & Sohn, Balingen, Germany). Their thickness and diameter were measured with a digital caliper (Vogel Germany GmbH & Co. KG, Kevelaer, Germany). The tablet hardness (resistance to breakage) was evaluated for 10 tablets (*n* = 10) using a tablet hardness tester (Erweka TBH 125D, Erweka, Langen, Germany). The results are expressed as the mean ± standard deviation.

Scanning Electron Microscopy

The morphology of tablet cross sections, produced using the SLS 3D printer and the Gamlen compaction simulator, was analyzed via scanning electron microscopy (SEM). To enhance conductivity during imaging, the samples were gold-sputtered at 30 mA for 100 s using the BAL-TEC SCD 005 device (Leica Microsystems, Wetzlaru, Germany). Micrographs were created using the JEOL JSM-6390LV scanning electron microscope (JEOL, Tokyo, Japan) at appropriate magnifications.

Fourier Transform Infrared Spectroscopy (FTIR)

FTIR spectroscopy was conducted to identify potential intermolecular interactions. The spectra of pulverized SLS tablets, pure atomoxetine, and individual excipients were recorded using the Nicolet iS10 spectrometer (Thermo Scientific, Waltham, MA, USA), equipped with a horizontal attenuated total reflectance (ATR) system (Smart iTR, Thermo Scientific, Waltham, MA, USA) and a zinc selenide lens. The spectra of the analyzed samples were recorded in the range of 4000 to 650 cm^−1^ with a resolution of 2 cm^−1^.

Differential Scanning Calorimetry (DSC)

The differential scanning calorimetry (DSC) method was employed to assess the physical state (crystalline or amorphous) of atomoxetine in the prepared physical mixtures and SLS tablets. The analysis was performed using a DSC1 device (Mettler Toledo, Giessen, Germany). Measured samples, consisting of 5 mg to 10 mg of pure atomoxetine and pulverized SLS tablets, were placed in aluminum pans and heated from 25 °C to 200 °C at a rate of 10°C/min under a constant nitrogen gas flow of 50 mL/min. Temperature and energy values were calibrated using an indium standard, while an empty aluminum pan served as a reference in all analyses. The obtained data were further analyzed using STARe software (version 12.10, Mettler Toledo).

X-Ray Powder Diffraction (XRPD)

XRPD analysis of SLS tablets was performed using an Empyrean diffractometer from Malvern Panalytical, Brighton, UK at room temperature, in Bragg–Brentano θ-2θ geometry, using a Cu-Kα radiation source with a nickel filter. Samples were scanned over a 2θ angle range of 4° to 70°, with a step size of 0.03° and a dwell time of 58 s per step, at a voltage of 45 kV and a current of 40 mA.

Disintegration

The disintegration analysis was performed using the Erweka ZT 52 disintegration tester (Erweka, Langen, Germany) with the use of plastic disks. The test was conducted in 800 mL of purified water as the medium, heated to 37 ± 0.5 °C. The disintegration of tablets produced by SLS 3D printing and the Gamlen compaction simulator was determined on 6 tablets of each formulation.

Determination of Atomoxetine Content in Tablets

The atomoxetine content in tablets produced using the SLS 3D printer and the Gamlen compaction simulator was determined spectrophotometrically with an Evolution 300 spectrophotometer (Thermo Fisher Scientific, Altrincham, UK) at a wavelength of 270 nm. To prepare the standard solution, 10 mg of atomoxetine was dissolved in 100 mL of distilled water. The solution was mixed in an ultrasonic bath (Bandelin Sonorex RK102H, Sonorex-Bandelin, Berlin, Germany) for 15 min at room temperature, then cooled and filtered into a beaker. For the test solution, three tablets from each formulation were crushed using a mortar and pestle. A mass equivalent to the average tablet weight was weighed on an analytical balance, transferred to a volumetric flask, and diluted with distilled water to 100 mL. The sample was then processed following the same procedure as the standard solution. The atomoxetine concentration in all formulations was determined using the standard solution of a known concentration.

Testing the Release Rate of Atomoxetine

The atomoxetine release rate from SLS 3D tablets and tablets produced on the Gamlen compaction simulator was evaluated using a USP II apparatus with a rotating paddle (Erweka DT600, Erweka, Langen, Germany). The dissolution medium consisted of 500 mL of distilled water, maintained at 37 ± 0.5°C. The paddle rotation speed was set to 50 rpm. Samples (5 mL) were collected at predefined time intervals (15, 30, 45, and 60 min) and subsequently filtered.

The atomoxetine content was determined spectrophotometrically at the maximum absorption wavelength of atomoxetine (270 nm) using the Evolution 300 spectrophotometer (Thermo Fisher Scientific, Altrincham, UK). Using the standard solution of a known concentration, the atomoxetine concentration was determined for each time point. The test was conducted in triplicate, and the values were presented as the mean percentage of dissolved drug substance (%) with the standard deviation.

AI-Assisted Tools

During the manuscript preparation, ChatGPT-4o (OpenAI, San Francisco, CA, USA) was utilized to verify the correctness of citations in accordance with the journal’s referencing style and to assess the adequacy of the English translation.

## 3. Results and Discussion

### 3.1. SLS Tablet Production

The published studies predominantly focus on the temperatures applied during tablet printing for achieving successful printing, but the specific methodology for selecting chamber and powder temperatures remains largely unexplored. In the SLS printing process, as well as in other 3D printing techniques, a major challenge lies in identifying and defining the optimal parameters that will ensure a successful printing process. The literature suggests that, for the SLS technique, it is desirable for the powder temperature to be at least 3 °C to 4 °C lower than the melting temperature of the mixture components or close to the glass transition temperature [27]. Furthermore, the chamber temperature should be lower than the powder surface temperature [26].

**Figure 1:**
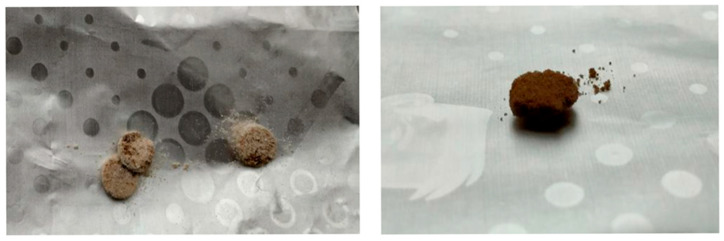
Examples of unsuccessful attempts at tablet printing using SLS 3D technique.

During the production of atomoxetine tablets using the SLS technique, the influence of parameters on the success of printing and the selection of suitable formulations and printing parameters were investigated through a trial-and-error approach. Printing parameters were deemed inadequate when the resulting tablets exhibited irregular shapes and/or colors that deviated from those of the physical powder mixture. Additionally, printing parameters were deemed inadequate in cases where the resulting tablets crumbled or disintegrated upon removal from the chamber. Some of the unsuccessful printing attempts are shown in Figure 1.

In formulations with co-processed excipients Retalac^®^ and Starlac^®^, the mixtures could not be considered printable, as in some cases it was not possible to produce a tablet, while in others, the resulting SLS tablets disintegrated during removal from the chamber due to inadequate mechanical properties. Additionally, the mixtures themselves expanded in volume during printing, which could be explained by the presence of lactose hydrates in each of the mentioned co-processed excipients. At the high chamber temperatures, the bound water evaporated, interfering with the sintering of powder particles in the mixture.

Subsequently, HPMC was used as an excipient, as its melting point (160 °C) falls within the operating temperature range of the SLS printer. It was found to be a suitable excipient for SLS printing, as successful tablet printing was achieved. Further attempts were then focused on determining the optimal printing parameters, namely the laser speed and chamber temperature. However, an issue occasionally occurred where the roller picked up too much material when applying a new layer. To address this, the lubricant sodium stearyl fumarate was removed from the final formulation.

The experimental results indicated that the optimal printing conditions for this formulation included a chamber temperature of 140 °C and a powder surface temperature of 150 °C. The laser speed was then varied, and it was concluded that tablets were successfully printed at laser speeds below 150 mm/s. Higher laser speeds did not result in successful printing, likely because larger tablets require greater energy for sintering. This implies that at higher laser speeds, the tablets did not receive sufficient energy from the laser.

**Figure 2:**
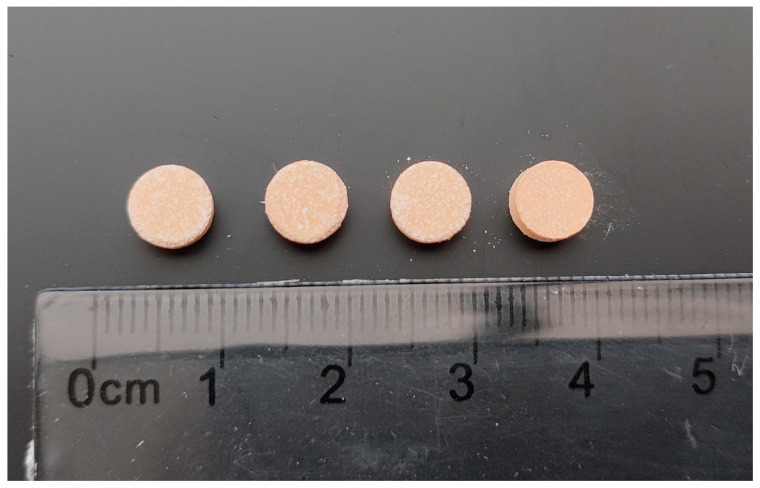
Manufactured SLS tablets of atomoxetine with dimensions 8 mm × 3 mm.

After identifying the successful printing parameters, the tablet printing process was carried out. Figure 2 shows atomoxetine tablets produced using the SLS technique, with dimensions of 8 mm × 3 mm. During a single cycle, 10 tablets were printed, and the time required for each cycle ranged from 10 to 20 min, depending on the tablet dimensions and the selected laser speed.

### 3.2. Tablet Production Using the Compaction Simulator

After preliminary experiments that defined the formulation composition and process parameters for successful SLS printing, tablets were produced using the same mixture containing 10% atomoxetine, 27% mannitol, 3% Candurin^®^ Gold Sheen, and 60% HPMC, with the Gamlen compaction simulator.

**Figure 3:**
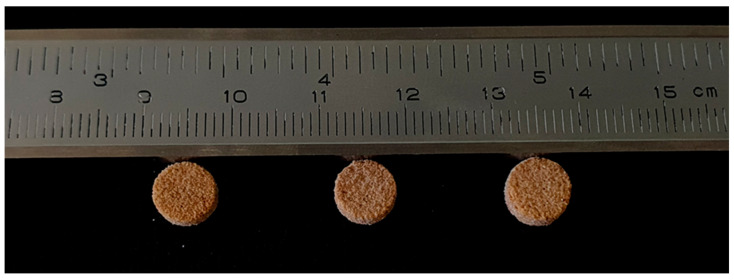
Tablets produced using a compaction simulator from the same physical mixture as the tablets printed using the SLS technique.

The produced tablets (Figure 3) were round, with a diameter of 6 mm and an average weight of 100 mg.

### 3.3. Tablet Characterization Results

Physical and Mechanical Properties

The physical and mechanical characteristics, disintegration time, and drug content of atomoxetine tablets produced using the SLS technique with different laser scanning speeds (from 100 to 200 mm/s), as well as those obtained with the compaction simulator, are presented in Table 2.

The data indicate that tablets printed at lower laser speeds exhibit a greater mass. This is attributed to the higher energy density applied during printing, which reduces the void spaces between powder particles and enhances particle sintering [28]. The measured diameter and thickness were close to the theoretical values, meaning that different printing parameters did not affect the dimensions of the produced tablets. The hardness of the SLS tablets and their disintegration time were directly correlated with the applied laser speed: as the laser speed increased, the tablets became less hard and disintegrated more quickly, due to the lower energy density and slightly weaker sintering of the powder particles.

Tablets produced using the Gamlen compaction simulator exhibited mechanical properties most similar to those of SLS tablets printed at a laser speed of 150 mm/s.

The disintegration time of the SLS 3D tablets ranged from 120 to 420 s, depending on the dimensions and laser speed applied during printing, while the disintegration time for the tablets produced using the Gamlen compaction simulator was 360 s. A positive effect of energy density on disintegration time was observed. Specifically, as the laser speed during printing increases, the sintering process becomes less intensive, resulting in easier particle separation within the tablets and consequently faster disintegration [28]. Importantly, all the tested formulations, regardless of the laser speed, met the disintegration requirements of the European Pharmacopoeia for uncoated tablets (NMT 15 min). This validates the robustness of SLS as a viable manufacturing method for immediate-release oral dosage forms and suggests that disintegration time can be modulated through process tuning, without altering the formulation composition.

Similar disintegration behavior was observed by Mohamed et al. when studying SLS tablets containing clindamycin [29]. The reference formulation compressed using the Gamlen compaction simulator exhibited disintegration behavior (360 s) most similar to SLS tablets printed at 150 mm/s, suggesting that mid-range laser scanning speeds may replicate the porosity and mechanical resistance typically achieved via conventional direct compression. This observation supports the potential of SLS to emulate traditional tablet attributes while offering greater design flexibility for tailored drug release profiles. Overall, these results demonstrate that SLS enables precise control over tablet disintegration by modulating energy input through laser speed and selecting thermally compatible excipients, providing a solid foundation for the development of patient-specific dosage forms with predictable in vitro performance.

**Figure 4:**
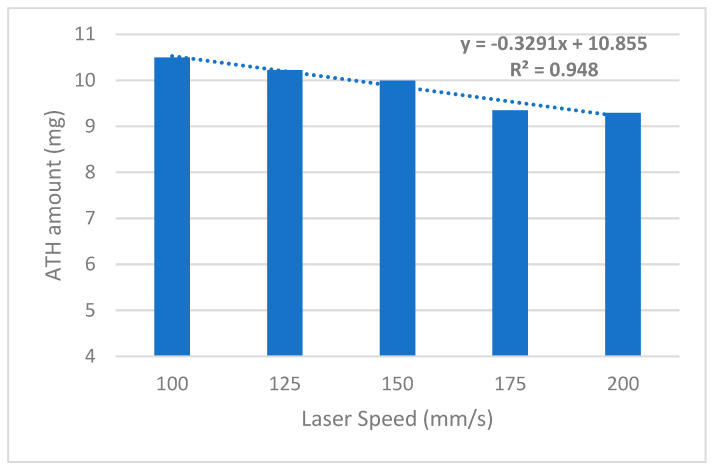
Average amount of incorporated atomoxetine in tablets fabricated with different laser scanning speeds.

The atomoxetine content in all tablets, produced using either the SLS printer or the Gamlen compaction simulator, ranged between 90% and 110% of the theoretical content. This confirms that no degradation of the drug substance occurred during either printing or compaction. The impact of varying the laser scanning speeds on the incorporated drug amount was further evaluated by calculating the absolute amount of atomoxetine in the fabricated tablets. All formulations were based on an identical powder mixture, with the laser scanning speed the only process parameter that varied. Interestingly, a clear inverse linear correlation was observed between the scanning speed and the amount of active substance incorporated into the final dosage forms (R^2^ = 0.948, Figure 4).

This finding confirms that, within the tested range, adjusting the laser scanning speed allows for precise modulation of the delivered drug dose, without the need for reformulating the powder blend. For instance, tablets printed at a speed of 100 mm/s incorporated approximately 10.50 mg of atomoxetine, while those fabricated at 200 mm/s contained 9.29 mg, despite identical design dimensions. Such control over the final dose through a single process parameter aligns with previous studies reporting the influence of the scanning speed on the printlet density and drug loading [30]. Although the observed absolute difference in dose may appear modest, it is important to emphasize that even small variations in drug content may have critical clinical implications, particularly in the case of highly potent compounds with a narrow therapeutic index (TI). Moreover, it is reasonable to expect that further increases in laser speed beyond the tested range would result in continued reduction in the incorporated dose, highlighting the importance of tightly controlled process parameters for precision dosing.

Importantly, this strategy directly addresses a key challenge in individualized therapy, the ability to prepare customized doses from a single, unified formulation, solely by modifying one process variable. In addition to influencing the drug content, the laser scanning speed also affected the disintegration time and drug release rate, indicating that both the dose and the bioavailability profile of SLS tablets can be tailored simultaneously. This modulation capability is highly desirable in personalized medicine, where individual patients may require not only different doses but also different pharmacokinetic profiles.

Nevertheless, the study also highlights a persistent limitation of current SLS-based processes: the optimization of printing conditions still relies heavily on the trial-and-error approach, lacking standardized predictive tools or regulatory guidelines. In this context, the observed dose–parameter relationship represents an important step toward rational process control, but further work is needed to validate this correlation across broader dose ranges, different active substances and individual patient needs.

Establishing robust process optimization frameworks and developing regulatory guidance specific to 3D-printed pharmaceuticals will be critical for translating these promising findings into clinical practice. Until then, each application will continue to require empirical optimization, which may limit the scalability and reproducibility.

Scanning Electron Microscopy

**Figure 5:**
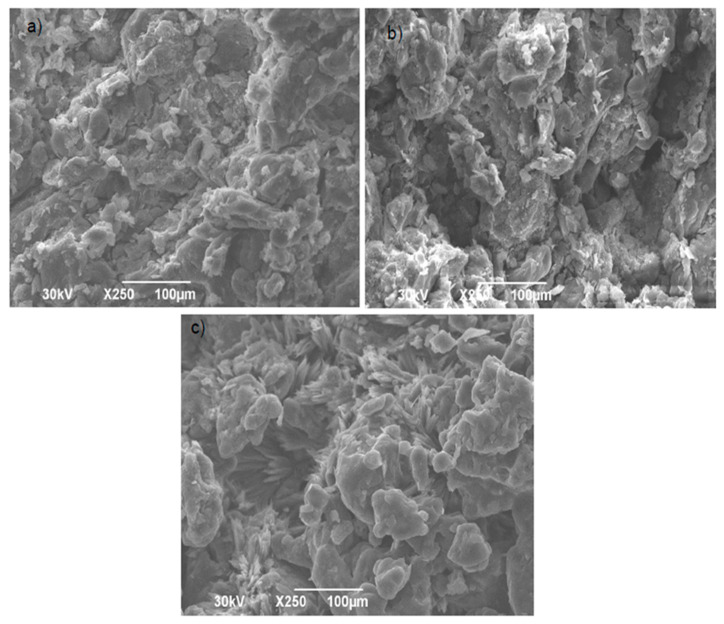
SEM micrographs of the cross section of atomoxetine tablets manufactured using (**a**) SLS technique at a laser speed of 100 mm/s; (**b**) SLS technique at a laser speed of 200 mm/s; (**c**) Gamlen compaction simulator.

SEM micrographs (Figure 5) reveal the morphological characteristics of tablet cross sections produced using the SLS 3D printer and the Gamlen compaction simulator.

In SLS tablets, certain segments were fully sintered, while others retained a powder-like structure, particularly at a laser speed of 200 mm/s. The resulting tablets have interparticle pores, which allow for rapid liquid penetration, leading to faster disintegration of the tablets.

These findings are consistent with the literature, which indicates that higher laser speeds and reduced energy density limit the extent of sintering, promoting the formation of interparticle pores [28]. SEM micrographs of tablets produced using the Gamlen compaction simulator show the presence of mesopores and macropores. Lower compaction forces, such as those that can be simulated using the Gamlen compaction simulator, have been shown to result in tablets with increased overall porosity. This includes the formation of both mesopores (2–50 nm) and macropores (>50 nm), which significantly influence the liquid penetration, disintegration, and drug-release characteristics [31].

Fourier Transform Infrared Spectroscopy (FTIR)

**Figure 6:**
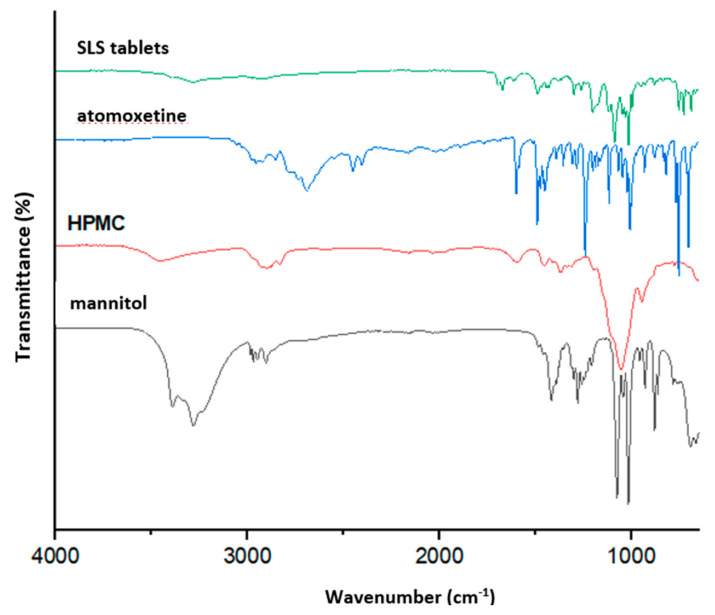
FTIR spectra of atomoxetine, excipients, and SLS-printed tablets.

The FTIR spectra of the starting materials (atomoxetine, HPMC, mannitol) and the produced SLS tablets are shown in Figure 6. The FTIR spectrum of HPMC exhibits absorption bands at 3450 cm^−1^ (OH stretching) and 2960 cm^−1^ (C-H stretching) [32]. A prominent band at 1060 cm^−1^ corresponds to out-of-phase vibrations of alkyl-substituted cyclic rings with ether bonds [33,34].The spectrum of mannitol displayed characteristic absorption bands at 3400 cm^−1^ (OH group stretching), 2947 cm^−1^ (C-H stretching), 1053 cm^−1^, and 1070 cm^−1^ (C-O stretching), consistent with the literature data [35]. The characteristic absorption peaks of atomoxetine at 1730 cm^−1^, 1600 cm^−1^, 1490 cm^−1^, 1475 cm^−1^, 1452 cm^−1^, 1356 cm^−1^, 1241 cm^−1^, 1202 cm^−1^, 1174 cm^−1^, 1062 cm^−1^, 1043 cm^−1^, 1008 cm^−1^, 991 cm^−1^, 820 cm^−1^, 768 cm^−1^, 754 cm^−1^, and 703 cm^−1^ can be observed at the same frequencies in both the FTIR spectra of pure atomoxetine and the FTIR spectra of the produced tablets.

The absence of shifts in these characteristic peaks confirms that no intermolecular interactions occurred between the drug substance and the excipients in the produced tablets.

Differential Scanning Calorimetry (DSC)

**Figure 7:**
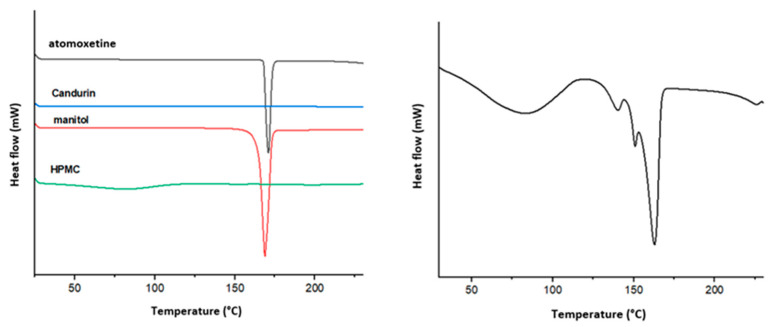
DSC thermograms: formulation components before printing (left) and 3D tablets produced using an SLS printer (right).

DSC analysis was conducted to determine whether the production of SLS tablets, under elevated temperatures and laser energy, altered the physical state of the drug substance. The DSC thermograms of the starting materials and the produced SLS 3D tablets are shown in Figure 7.

The DSC thermogram of pure atomoxetine shows a sharp endothermic peak at 169 °C, corresponding to the melting of the crystalline drug substance. Additionally, a sharp endothermic peak at 168 °C was observed, corresponding to the melting point of mannitol.

The thermogram of the SLS-produced tablets shows the characteristic endothermic peaks of atomoxetine and mannitol, confirming that both substances retained their crystalline state despite exposure to elevated temperatures and laser energy. On the thermogram of HPMC, a broad endotherm is observed in the range of 50 °C to 80 °C, corresponding to the evaporation of loosely bound or absorbed water. The evaporation of water was expected for the raw substance due to the large surface area of the particles.

In the same temperature range, an endothermic peak is also observed on the thermogram of SLS-printed tablets, which further indicates significant water evaporation during heating in the testing process. This can be explained by the complex internal structure of 3D-printed objects. The samples were further analyzed using X-ray powder diffraction (XRPD) to confirm the results of the DSC analysis.

X-Ray Powder Diffraction (XRPD)

XRPD analysis was performed to gain a more detailed understanding and confirm the physical state of the drug substance in the produced SLS 3D tablets.

**Figure 8:**
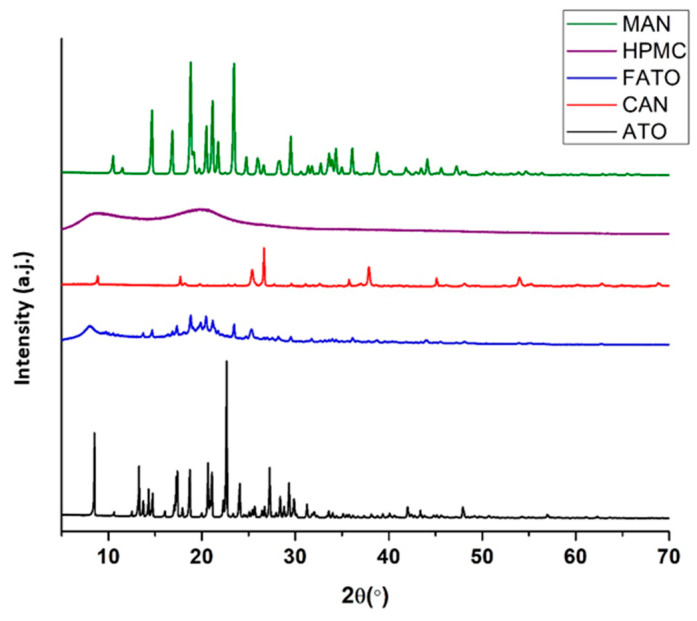
Diffractograms of the formulation components and SLS 3D-printed atomoxetine tablets produced at a laser speed of 150 mm/s.

The diffractograms obtained from the analysis of atomoxetine, mannitol, HPMC, Candurin Gold Sheen, and selected SLS 3D tablets are shown in Figure 8. The X-ray diffractogram of atomoxetine exhibits the characteristic pattern of crystalline substances, as evidenced by the presence of numerous sharp and intense peaks.

The atomoxetine diffractogram displays characteristic high-intensity peaks at 17.6°, 18.2°, 18.9°, 21.3°, 23°, 24.3°, and 27.5° 2θ. Based on the positions of these characteristic peaks, the atomoxetine diffractogram corresponds to the diffractogram of its crystalline form as described in the literature [36].

The presence of these characteristic peaks is also observed in the diffractogram of the produced SLS tablets. This confirms that the drug substance is present in its crystalline form in the produced SLS tablets, which correlates with the results of the DSC analysis.

Both the DSC and XRPD analyses confirmed that the crystalline state of atomoxetine was retained in the SLS-printed tablets. However, additional studies are needed to investigate the long-term stability of these formulations, particularly considering their exposure to localized thermal energy during printing. The stability protocols defined by the International Council for Harmonisation of Technical Requirements for Pharmaceuticals for Human Use (ICH) for stability testing of conventional dosage forms have long been established as the standard for ensuring product quality over time. Nonetheless, their direct applicability to 3D-printed medicines remains uncertain, and regulatory pathways for assessing the stability of such dosage forms are yet to be clearly defined.

Testing the release rate of atomoxetine

**Figure 9:**
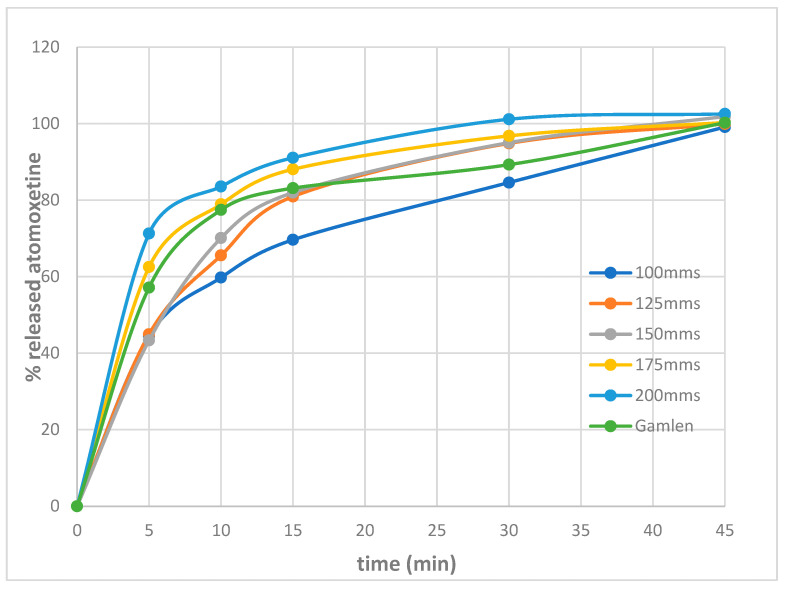
Graphical representation of the release rate of atomoxetine from tablets produced using an SLS printer at different laser speeds and tablets manufactured using the Gamlen compaction simulator.

Figure 9 shows the release profiles of atomoxetine from tablets produced using the SLS printer and the compaction simulator, with varying laser speeds applied for the SLS tablets.

From the presented results, it can be concluded that all the tablets, both the SLS 3D tablets produced with varying laser speeds and the tablets produced using the Gamlen compaction simulator, achieved complete and full release of atomoxetine within 45 min. This meets the requirements of Chapter 5.17.1. Recommendation on Dissolution Testing of the European Pharmacopoeia, edition 11.0, regarding pharmaceutical forms with conventional drug release. The acceptance criterion at the S1 level specifies that at least 80% of the drug substance should be released within the defined time, typically 45 min or less.

Moreover, SLS tablets printed at laser speeds of 175 mm/s and 200 mm/s exhibited ultra-rapid release, with over 85% of atomoxetine released within 15 min. As previously noted, increasing the laser speed during printing reduces the intensity of the sintering process, allowing particles within the tablets to separate more easily. This results in faster tablet disintegration, faster contact with the dissolution medium, and consequently, faster dissolution of the drug substance. This finding correlates with the observed shorter disintegration times at these laser settings, suggesting that increased tablet porosity due to lower sintering density promotes faster penetration of the dissolution medium and more efficient drug release. Conversely, tablets printed at lower laser speeds (e.g., 100 mm/s) disintegrated more slowly and demonstrated a more gradual release profile, consistent with their denser microstructure and longer disintegration times.

Given that the tablets produced using the Gamlen compaction simulator exhibited mechanical properties most similar to SLS tablets printed at a laser speed of 150 mm/s, their dissolution profiles were compared using the similarity factor (f2).The calculated similarity factor (f2) was 80.17, exceeding the threshold of 50. This indicates that the atomoxetine release profiles from the SLS tablets printed at a laser speed of 150 mm/s and the tablets produced on the Gamlen compaction simulator are highly comparable. These findings demonstrate a clear link between laser scanning speed, tablet disintegration, and drug release kinetics, highlighting the potential of SLS technology to tailor the dissolution performance of tablets by fine-tuning the process parameters, without altering the formulation composition.

## 4. Conclusions

This study demonstrates that the selective laser sintering (SLS) technique can be effectively utilized to produce tablets with satisfactory mechanical and pharmaceutical-technological properties. By optimizing key process parameters, such as the chamber and powder surface temperatures and the laser speed, the SLS process was successfully adapted for the production of atomoxetine tablets.

The incorporation of hydroxypropyl methylcellulose (HPMC) as the primary polymer in the formulation proved to be a critical factor in ensuring successful tablet printing. The SLS-produced tablets exhibited physical and mechanical characteristics comparable to those manufactured under industrial conditions via the direct compression process using a Gamlen compaction simulator. Furthermore, complete drug release was achieved within 45 min for both methods, meeting the standards outlined in the European Pharmacopoeia. Notably, SLS tablets printed at higher laser speeds (175–200 mm/s) demonstrated ultra-rapid drug release, with over 85% of atomoxetine released within 15 min, highlighting the flexibility of this technique in tailoring drug release profiles.

The findings from the XRPD, FTIR, and DSC analyses confirmed the retention of the crystalline structure of atomoxetine and the absence of any significant intermolecular interactions between the drug and excipients during the printing process. However, further studies are needed to evaluate the long-term stability of SLS-printed atomoxetine tablets and to align future testing with appropriate regulatory frameworks.

This study demonstrated that precise dose adjustment can be achieved by using a single formulation and varying only one process parameter, laser scanning speed. However, further work is needed to standardize the process optimization and move beyond trial-and-error toward regulatory adoption.

Overall, this research highlights the significant potential of SLS printing as a versatile and efficient method for producing tablets with customizable properties. The ability to produce tablets with comparable characteristics to those manufactured via conventional techniques underscores its applicability not only for personalized medicine but also for small-scale production. These results contribute to a deeper understanding of the SLS printing process and mark an important step toward optimizing and standardizing this technology for broader pharmaceutical applications.

## Figures and Tables

**Table 1:** Composition of the formulations and process parameters.

Composition of the Mixtures	Chamber/Surface Temperature (°C)	Laser Scanning Speed (mm/s)
ATH 10% Candurin 3% Sodium stearyl fumarate 2% Croscarmellose sodium 2% Kollidon^®^ 6% Starlac^®^ 76%	120/130 120/130 120/135 130/140 140/150 140/150	100 150 100 100 150 100 150
ATH 10% Candurin 3% Sodium stearyl fumarate 2% Croscarmellose sodium 2% Kollidon^®^ 6% Retalac^®^ 76%	120/130 120/130 120/135 130/140 140/150 140/150	100 150 100 100 150 100 150
ATH 10% Candurin 3% Sodium stearyl fumarate 2% Mannitol Partec 25% HPMC 60%	120/130	100
	130/140	100
	140/150	100
ATH 10% Candurin 3% Mannitol Partec 27% HPMC 60%	130/140 130/140 140/150 140/150 140/150 140/150	100 150 100 150 175 200

**Table 2:** Comparison of the characteristics between the SLS- and Gamlen-produced tablets.

Formulation	Weight (mg)	Diameter (mm)	Thickness (mm)	Hardness (N)	Disintegration Time (s)	Drug Content (%)	Drug Amount (mg)
Fsls-100	103 ± 0.91	7.97 ± 0.1	3.16 ± 0.1	45.5 ± 4.8	420	10.19	10.49
Fsls-125	101 ± 0.25	7.99 ± 0.1	3.15 ± 0.1	38.7 ± 5.6	380	10.12	10.22
Fsls-150	98.9 ± 0.74	7.99 ± 0.1	3.09 ± 0.1	22.6 ± 7.9	320	10.10	9.98
Fsls-175	94.5 ± 0.15	7.91 ± 0.1	3.16 ± 0.1	18.3 ± 2.5	200	9.89	9.35
Fsls-200	92.6 ± 0.11	7.93 ± 0.1	3.15 ± 0.1	14.5 ± 3.2	120	10.03	9.28
F-Gamlen	99.8 ± 0.4	6.00	3.00	24	360	10.00	9.98

## Data Availability

The data presented in this study are available on request.
